# Gender-specific analysis of the authors and the editorial board of *Naunyn–Schmiedeberg’s Archives of Pharmacology* from 2000 to 2020

**DOI:** 10.1007/s00210-021-02166-3

**Published:** 2021-10-08

**Authors:** Rebecca Zehetbauer, Florentin von Haugwitz, Roland Seifert

**Affiliations:** 1 Institute of Pharmacology, Hannover Medical School, Carl-Neuberg-Str. 1, 30625 Hannover, Germany; 2 Nova School of Business and Economics, Carcavelos Campus, Rua da Holanda, no. 1, 2775-405 Carcavelos, Portugal

**Keywords:** Gender structure, *Naunyn–Schmiedeberg’s-Archives of Pharmacology*, Female quota, Editorial board, Authors

## Abstract

Motivated by the worldwide debate on gender equality, we analyzed the gender structure of the authors and the editorial board of *Naunyn–Schmiedeberg’s Archives of Pharmacology*. We wrote an algorithm to assign authors’ first names to a gender to determine the gender distribution of publications. We evaluated publications from German research institutes from 2000 to 2020. This resulted in a data set of 2929 authors. We could assign a first name to almost all authors. The percentage of female authors increased until 2008, but thereafter stagnated at around 30%. The position of senior author is far less often held by women (around 15%). Even though multiple political measures were implemented to increase the female participation, our analysis has shown no increase regardless. We also observed a strong decrease in German authorship (both male and female) since 2000. In the editorial board and for advisory editors, there has been a substantial increase in the proportion of women since 2016 as the result of appointments by the editor-in-chief. We discuss the strengths and limitations of our study in context with the literature and current developments in society and science and methodological pitfalls of studies in this field. More research is required to obtain a full picture of gender structure in science and to be able to properly interpret the data.

**Supplementary Information:**

The online version contains supplementary material available at 10.1007/s00210-021-02166-3.

## Introduction

With its first issue in 1873, *Naunyn–Schmiedeberg’s Archives of Pharmacology* is the oldest journal of pharmacology still in existence (https://www.springer.com/journal/210, last accessed on August 2, 2021). Motivated by the current global debate in politics and society about gender equality, we wished to analyze the gender structure of authors and the editorial board in this journal. The long-term goal is to draw conclusions about the *status quo* of gender equity in pharmacological research, in particular, and biomedical research more generally.

Different aspects of society are increasingly examined for gender equity and discrepancies. For example, in an editorial, *Nature* examined the gender structure of its authors (Nature. 2018 Jun;558(7710):344. https://doi.org/10.1038/d41586-018-05465-7. PMID: 29,925,975). The percentage of women authors was around 30%, and the percentage of women in the position of corresponding author (senior author) amounted to 16%, demonstrating *Natur*e’s under-representation of women. *Nature* examined around 900 authors over a period of 1 year in different sections of the journal. *Nature* had a hard time clearly assigning a gender to a name in the case of unisex names from countries like China, which is why these results are probably biased.

Compared to the *Nature* analysis, we focused only on one scientific field, i.e., pharmacology, and analyzed *Naunyn–Schmiedeberg’s Archives of Pharmacology* in a period of 21 years instead of just 1 year. This is particularly relevant because the time since 2000 was characterized by a social rethinking (https://www.zukunftsinstitut.de/artikel/die-zukunft-ist-weiblich-megatrend-female-shift/, last accessed on August 2, 2021). For this purpose, we examined 2929 authors from German research institutes. We chose Germany because German first names, with relatively few exceptions, can be unequivocally assigned to a female and male gender (https://www.vorname.com/das-sind-die-schoensten-unisex-vornamen.html; last accessed on August 2, 2021). Therefore, our analysis is not random sampling and not biased.

## Materials and methods

### Analysis of authors

To be able to study the evolution of the gender ratio in pharmacological research, we had to evaluate the gender of each researcher. For this purpose, all metadata of the papers published in *Naunyn–Schmiedeberg’s Archives of Pharmacology* were kindly made available to us by *Springer Nature* as an excel spread sheet. To accurately match first names to a gender, we ran our database through a large first name database (https://github.com/MatthiasWinkelmann/firstname-database/blob/master/firstnames.csv, last accessed on April 11, 2021) which assigned a gender to each first name. We knew that it would be difficult to clearly assign names from countries like China to a gender, as they are more commonly used for both men and women (https://sg.theasianparent.com/asian-gender-neutral-names, last accesses August 2, 2021).

Unfortunately, in a considerable number of names (> 15% of all authors) only the initials of the authors’ first names are provided, substantially complicating the evaluation. For this reason, we decided to limit ourselves to publications from Germany to prevent the problem of unisex first names already mentioned above. We filtered out all papers that had “Germany” in the “Country of Research Organization” column. We also decided to limit the time frame of our analysis to the years 2000–2020, including publications from January 2000 to December 2020. We wanted the analysis to be as comprehensive as possible, and with this time frame, we assumed that any Internet research would still be reasonably possible. At the same time, it was important to choose a sufficiently long time to be able to examine societal development. Since the last 20 years have been characterized by societal changes (https://www.bpb.de/apuz/144851/entgrenzungsdynamiken-geschlechterverhaeltnisse-im-umbruch, last accessed on August 2, 2021), this period seemed to be particularly interesting.

To analyze the papers and their authors as precisely and uniformly as possible, we used the software *pandas* (McKinney, Proceedings of the 9th Python in Science Conference, Volume 445, 2010 https://pandas.pydata.org) and the programming language *python* (Van Rossum, G., & Drake, F. L. (2009). *Python 3 Reference Manual*. Scotts Valley, CA: CreateSpace). We imported the data from *Springer Nature* and filtered them in accordance with our criteria. The publications with Germany as the country of research and a publication date within our time of interest were extracted, and the names of the authors listed in a table in the same order as on the publication. If the paper was a collaboration of several institutes from different countries, then only the researchers from German institutions were included. These steps resulted in a count of 2929 authors and 651 articles. These data show that in average, a given paper had 4.5 authors, rendering a differentiated analysis according to total number of authors, first author and senior author feasible. After we ran our data through the first name database, we were able to then link the first names of the authors to the chosen first name database. When a match was found, the gender of the author was added to our table.

The 522 first names that were abbreviated or could not be clearly assigned to a gender were flagged and then evaluated manually. The reason that some first names could not be clearly assigned to a gender is that researchers from all over the world also work at German pharmacological institutes and thus do not have European first names. However, to include these in our analysis as well, we searched *PubMed* (https://pubmed.ncbi.nlm.nih.gov, last accessed on June 25, 2021) and *Research Gate* (https://www.researchgate.net/?_sg=IW-0A5DIZeHggkJUVZ-1mekSXPTHcf-7pgwaWxXZ5AM6CRFyWvZ41M0Czh13lxNbUEfdNgSd5aCQ), last accessed on June 25, 2021) for the respective publications to determine the complete first name and thus the gender on the profile of the respective authors. If this was not possible, we searched for the researchers on the website of the respective institutes.

The Internet search became more difficult with each year going further into the past, since the research was not yet fully digitalized. Hence, we called the institute secretaries and asked for the respective researcher to determine their gender. In some cases, we were also able to identify the gender of the coauthors by contacting the corresponding author. In addition, the office of the *German Society for Experimental and Clinical Pharmacology and Toxicology* (DGPT) in Düsseldorf kindly provided their first and last name directory of past and present members (no other information was provided to ensure personal data protection). This list also helped us assign more names to female or male gender. Similarly helpful were the extensive works of Athineos Philippu on the history of pharmacology in German-speaking countries (Philippu, A. P. (Ed.). (2004–2021). *Geschichte und Wirken der pharmakologischen, klinisch-pharmakologischen und toxikologischen Institute im deutschsprachigen Raum*: Bd. I–VI (1. Aufl.). Berenkamp Verlag, Wattens, Austria) which contains a comprehensive index of names. In this way, we were able to reliably identify several emeritus researchers.

After this extensive multipronged research, the number of non-determinable names had shrunk from 522 to 42, giving us an evaluation rate of 98.57%. The reason for not being able to evaluate the remaining 42 authors may be that they married and changed names or could not be found on the Internet due to inactivity in research or death. Because the author names were transferred to our table in the same order as listed on the paper, the first name of each DOI was marked as first author, and the last name of each DOI was marked as senior author.

In extracting the data, we were also interested in the city where the research and paper came from. This also allowed us to examine regional differences in the development of the female quota within Germany. To obtain a representative result, we only examined cities with more than 200 authors in the period 2000–2020. The three cities meeting this requirement were Bonn (located in western Germany), Heidelberg (located in south-western Germany), and Hannover (located in central-northern Germany). Thus, a reasonable geographic representation of Germany was achieved.

### Analysis of the editorial board

To evaluate the editorial board members and the advisory editors, we could not access the online volumes of the *Naunyn–Schmiedeberg’s Archives of Pharmacology* because only the collection of the individual publications is available online, but the editorial board is listed on the first pages of a print edition. Therefore, we had to collect the print editions of the years 2000–2020 from private collections and from university libraries in Hannover and Munich and evaluate them manually.

Again, since the first names of the editorial board members as well as the advisory editors were abbreviated from 2000 to 2012, further research was necessary. In evaluating the editorial board, we did not limit ourselves to researchers from Germany, but evaluated names from all countries, because the editorial board is composed of researchers from many different countries to enhance the internationality and diversity of the journal. In fact, we had an evaluation rate of 100% because only researchers who are experienced and renowned in their respective research fields and subsequently leave a robust footprint on the Internet are appointed to editorial board positions. We separately evaluated the editorial board members and the advisory editors. The reason for this is that editorial board members edit submitted research papers independently, appoint reviewers for paper evaluation, and decide independently on the acceptance or rejection of a paper. Advisory editors are a core pool of reliable and proven reviewers who are called upon by the Editors for reviews.

## Results

### Almost complete author analysis

**Fig. 1:**
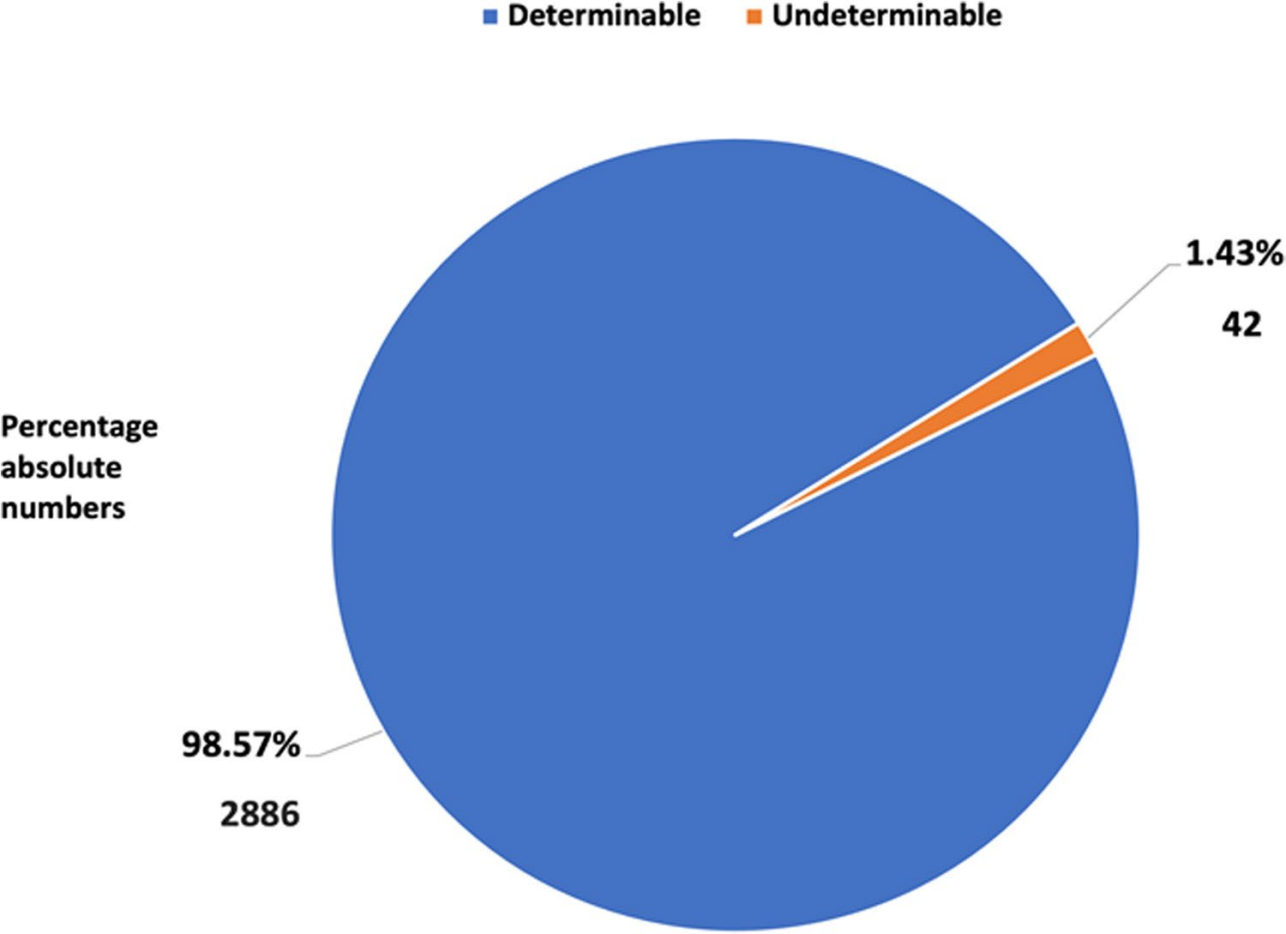
Evaluation quota of all authors from German research institutes from 2000 to 2020. Data are shown in a pie chart, with the determinable share in blue and the undeterminable share in orange

**Fig. 2:**
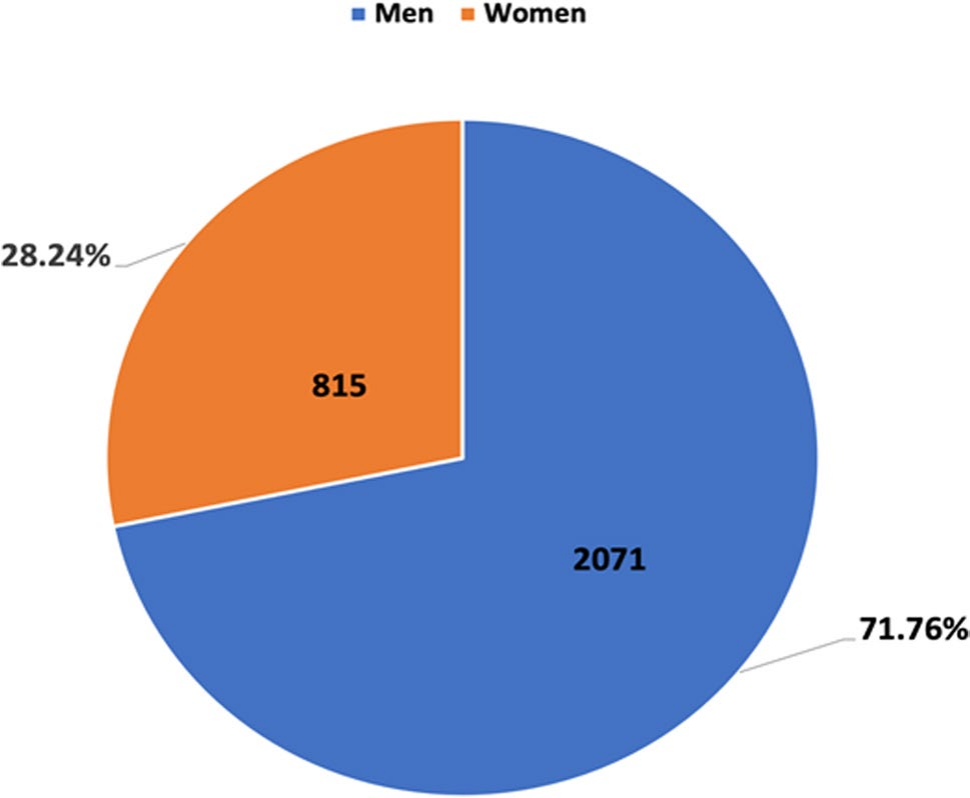
Gender ratio of all authors from 2000 to 2020. Data are shown in a pie chart, with the percentage of women in orange and the percentage of men in blue. Only the 2.886 evaluated authors are included

In 98.57% (2886) of the 2929 authors, we were able to determine the gender by the above-described methods. This was not possible for just 1.43%, i. e., 42 authors (Fig. 1). Reasons for this could be that these authors changed their last name due to marriage and that they became inactive in pharmacological research or died. Of the remaining 2886 authors from German research institutes over the last 21 years, 72% (2071) are men, and 28% (815) are women (Fig. 2). Thus, we started with an almost complete data set of clearly defined names.

### Analysis of total authorship

**Fig. 3:**
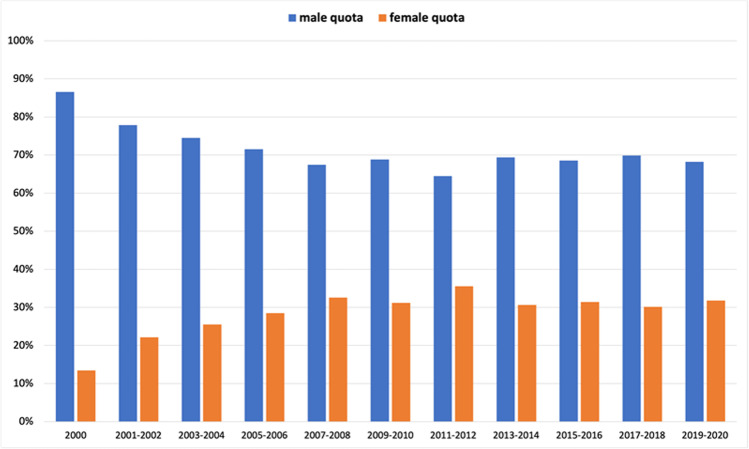
Gender ratio of all authors, presented in a 2(1)-year interval. Data are presented in a bar chart, with the percentage of women in orange and the percentage of men in blue

**Fig. 4:**
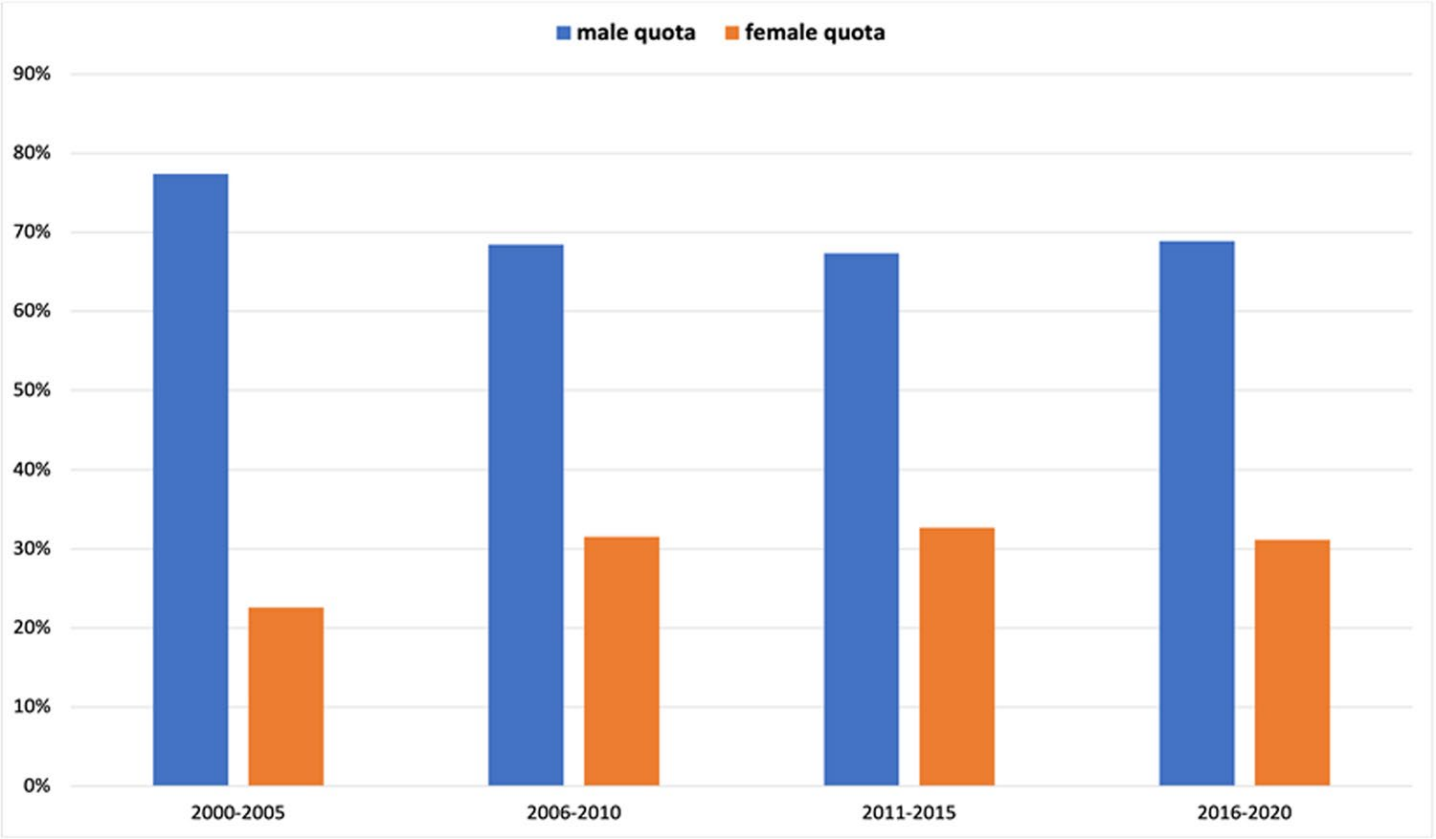
Gender ratio of all authors, presented in a 5(6)-year interval. Data are presented in a bar chart, with the percentage of women in orange and the percentage of men in blue

To analyze the evolution of the gender ratio over the past 21 years, we plotted the gender ratio in a 2-year interval (1-year interval for 2000) in a bar chart (Fig. 3). This showed a continuous increase in female authors over the period of 2000–2008 from 13 (26 out of 192) to 33% (84 out of 259). Thereafter, the percentage of women remained at around 30% after a maximum of 36% (105 out of 297) in 2011–2012. To show the trend more clearly without outliers, we also decided to create a graph with a 5-year interval (6-year interval for 2000–2005) (Fig. 4). This shows once again that there has been no significant change in the proportion of women over the last 10 years.

### Analysis of first authorship

**Fig. 5:**
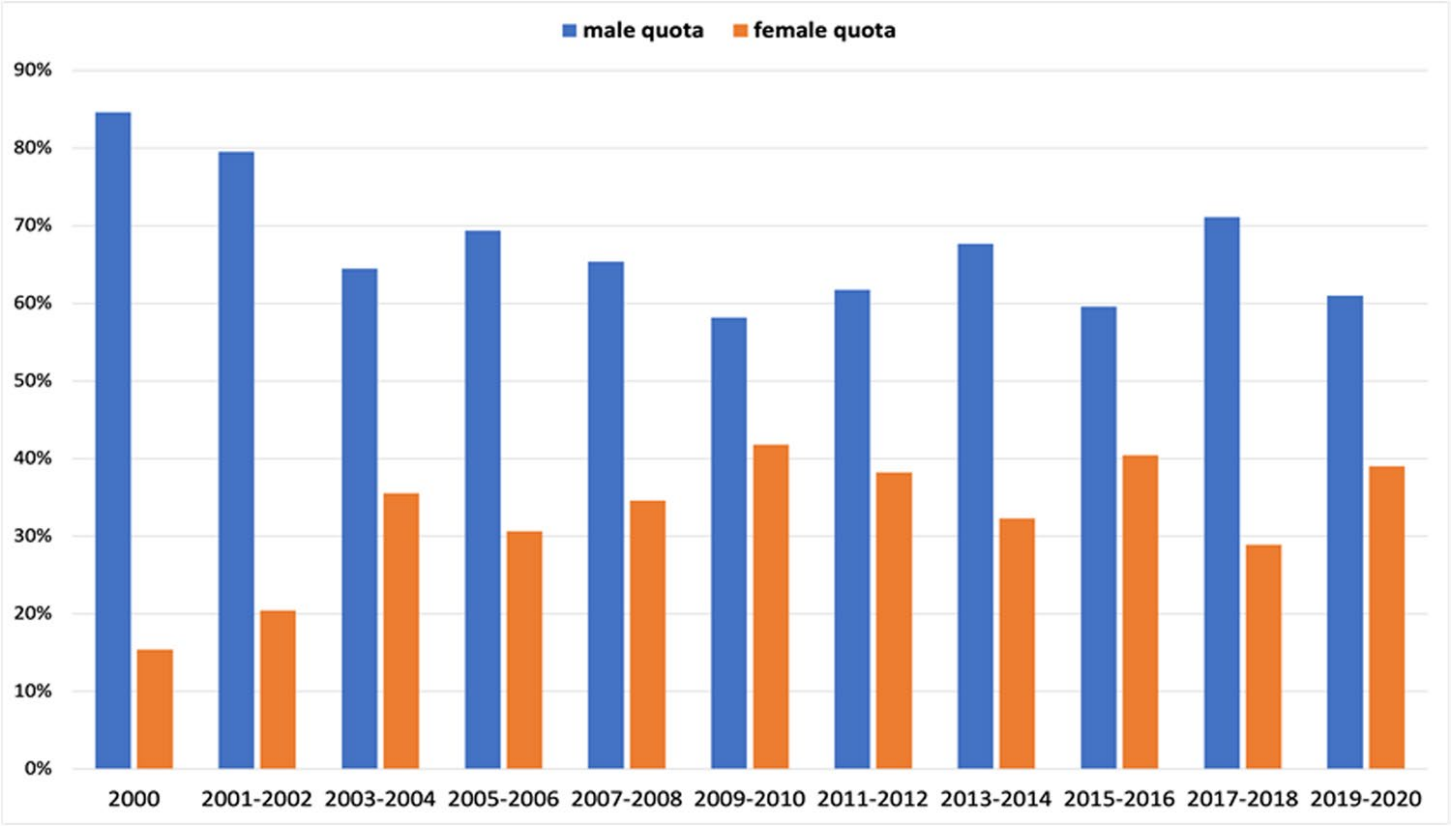
Gender quota of the first author, presented in a 2(1)-year interval. Data are presented in a bar chart, with the percentage of women in orange and the percentage of men in blue

**Fig. 6:**
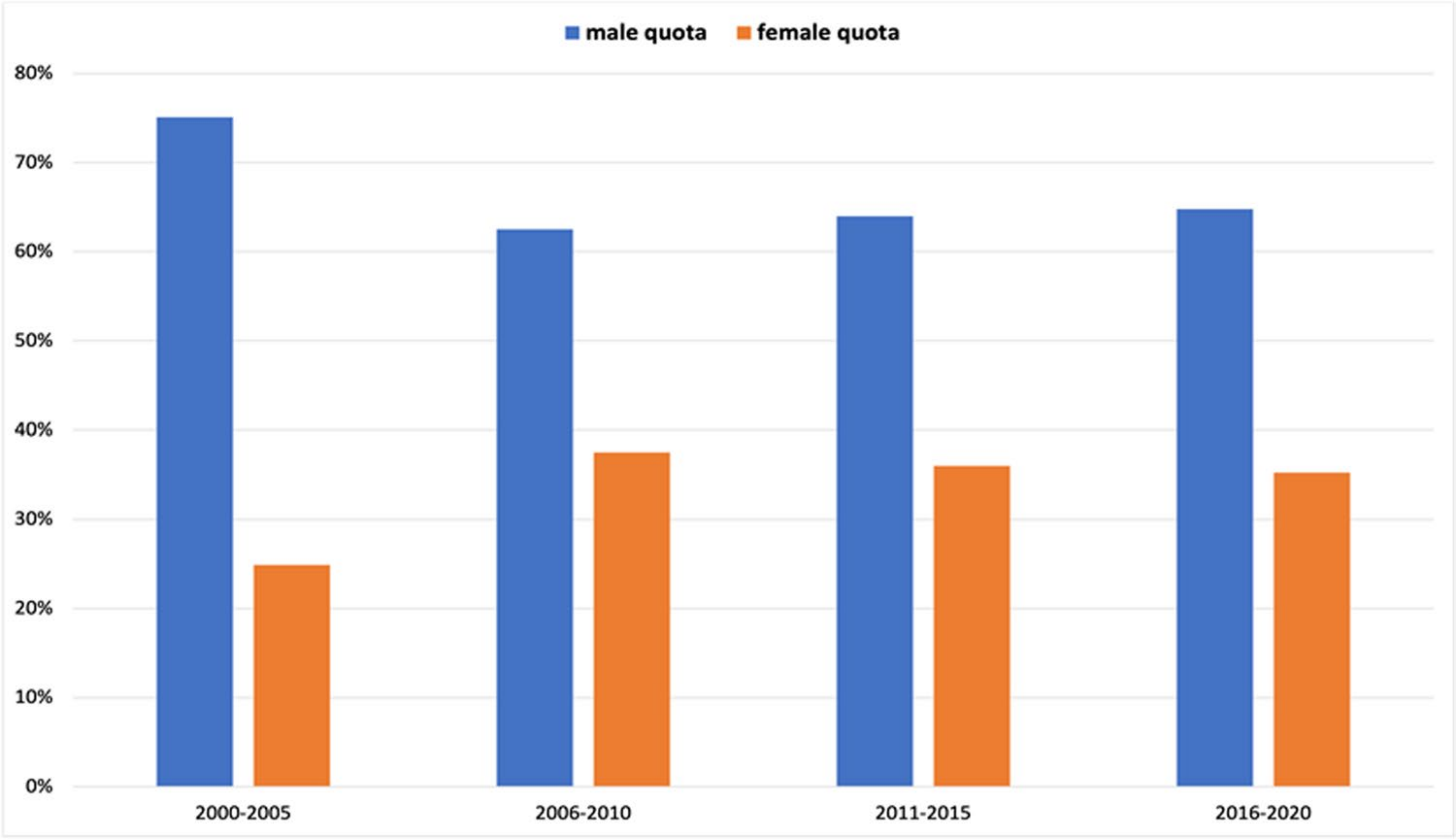
Gender quota of the first author, presented in a 5(6)-year interval. Data are presented in a bar chart, with the percentage of women in orange and the percentage of men in blue

We used the same presentation method to examine the gender structure of the first author positions. In the 2(1)-year interval analysis (Fig. 5), we found an increase in female first authors from 15 (8 out of 52 total authors) to 36% (27 out of 76) in 2000–2004. After dropping to 31% (19 out of 62) in 2005–2006, the percentage of women in this position increased to 42% (23 out of 55) in 2009–2010, after which it fell and rose again, amounting to 39% (16 out of 41) in the period 2019–2020. In the 5(6)-year interval analysis (Fig. 6), there is an increase in the female ratio from 25 (62 out of 249 total authors) in 2000–2005 to 38% (51 out of 136) in 2006–2010, which decreased to 36% (58 out of 161) in the following 5 years and then again to 35% (37 out of 105) in 2016–2020.

### Analysis of senior authorship

**Fig. 7:**
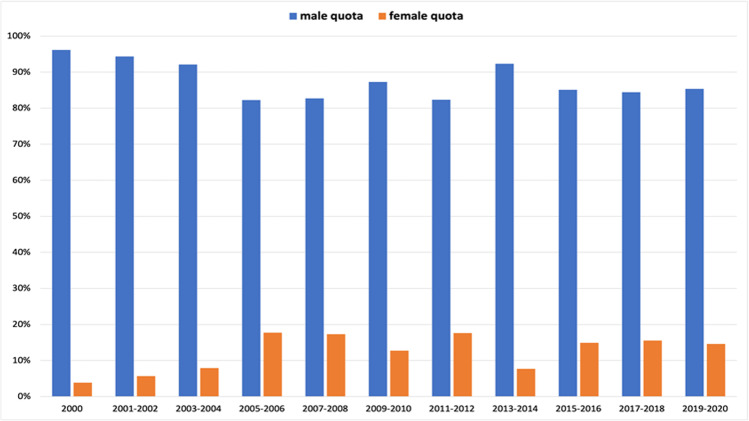
Gender quota of the senior author, presented in a 2(1)-year interval. Data are presented in a bar chart, with the percentage of women in orange and the percentage of men in blue

**Fig. 8:**
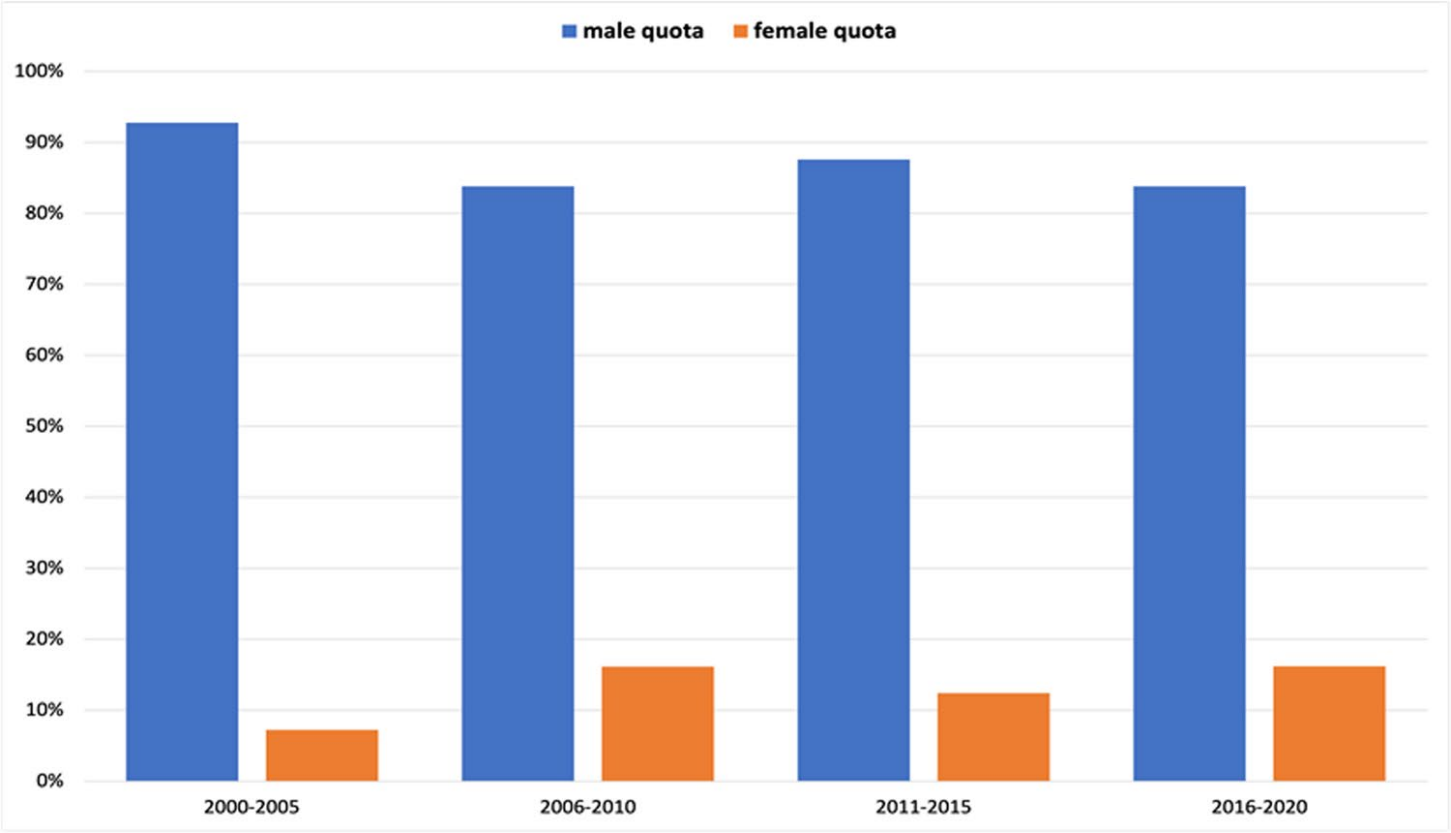
Gender quota of the senior author, presented in a 5(6)-year interval. Data are presented in a bar chart, with the percentage of women in orange and the percentage of men in blue

In senior author position, only 4% (2 out of 52 total authors) were women in 2000 (Fig. 7). This number increased to 18% (11 out of 62) in 2005–2006 and remained at about this level, interrupted by a drop to 13% (7 out of 55) in 2009–2010. Then, in 2013–2014, the percentage of women dropped to 8% (5 out of 65), levelling off to 15% in the following years. The 5(6)-year interval analysis shows an increase a female senior authorship from 7% (18 out of 249) in the period 2000–2005 to 16% (22 out of 136) in the following 5 years (Fig. 8). In 2011–2015, the percentage decreased to 12% (20 out of 161) and finally recovered to 16% (17 out of 105).

### Regional analysis of Germany

**Fig. 9:**
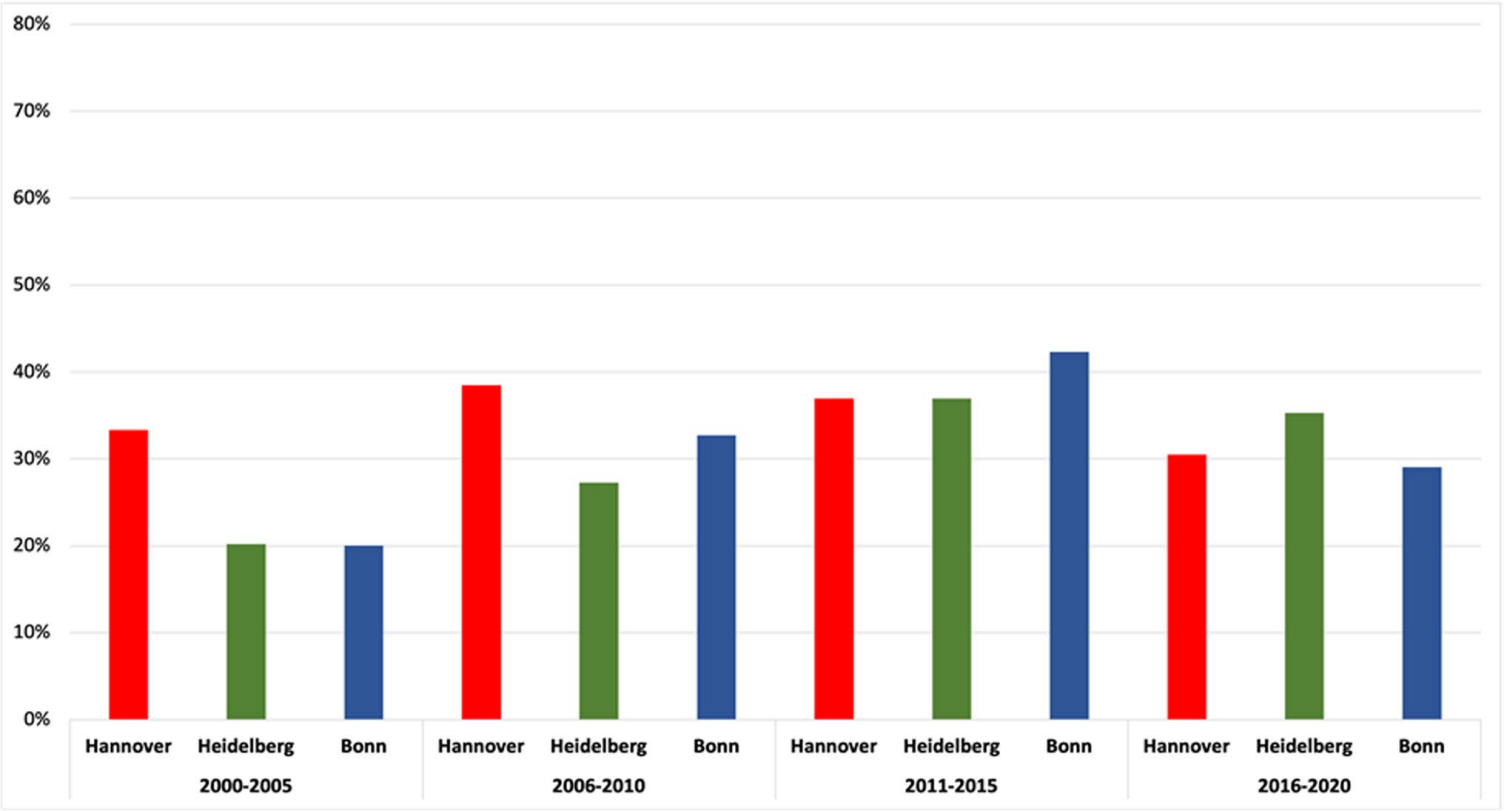
Gender quota of the research institutes in Hannover, Heidelberg and Bonn, presented in a 5(6)-year interval Data are presented in a bar chart, with the percentage of women in Hannover in red, the percentage of women in Heidelberg in green and the percentage of women in Bonn in blue

Hannover, Heidelberg, and Bonn are the cities in which more than 200 researchers each have been involved in papers published in *Naunyn–Schmiedeberg’s Archives of Pharmacology* from 2000 to 2021. We placed the evaluations of the individual cities side-by-side in a bar chart for comparison. Since we only wanted to determine the trend there, we decided to use a 5(6)-year interval analysis (Fig. 9). Hannover started with the highest percentage of female authors of 33% (1 out of 3) and increased this to 39% (15 out of 39) in the years 2006–2010. Thereafter, however, the percentage of women dropped to 31% (18 out of 59) in the period 2016–2020. Heidelberg started with a female quota of 20% (20 out of 99) in 2000–2005. It recorded an increase to 27% (27 out of 99) in 2006–2010 and then to 37% (34 out of 92) in 2011–2015. The proportion decreased to 35% (18 out of 51) in 2016–2020. With 20% (11 out of 55), Bonn had almost the same female quota as Heidelberg in 2000–2005, but this increased to 33% (18 out of 55) in the following 5 years and then to the peak of 42% (22 out of 52) in 2011–2015. However, the percentage of female authors then substantially decreased to 29% (9 out of 31) in 2016–2020.

### Decrease in the absolute number of authors from Germany

**Fig. 10:**
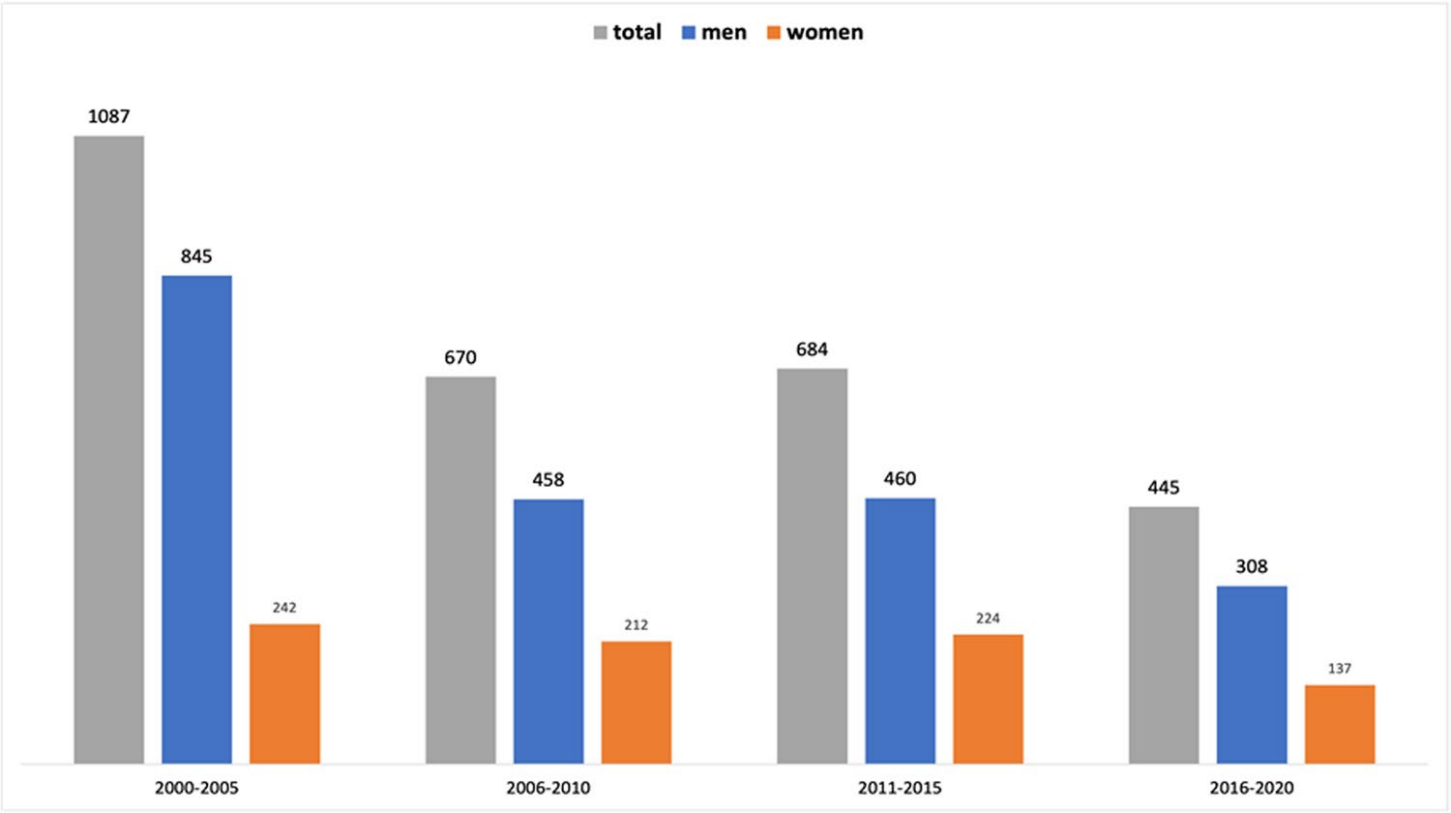
Gender share of all authors in absolute terms, presented in a 5(6)-year interval. Data are presented in a bar chart, with the absolute numbers of women in orange and the absolute number of men in blue. The total number of authors is shown in gray

The absolute number of authors also changed over the analyzed time (Fig. 10). In the time from 2000 to 2005, *Naunyn–Schmiedeberg’s Archives of Pharmacology* had close to 1100 authors with a female share of 22.2% (242 out of 1087). Within the next 10 years, the number of authors decreased by about 40% with a female share of around 32%. Within the last time analyzed, the number of publications had decreased by about 60% from the starting value with the female share hovering at around 30%.

### Editorial board analysis

**Fig. 11:**
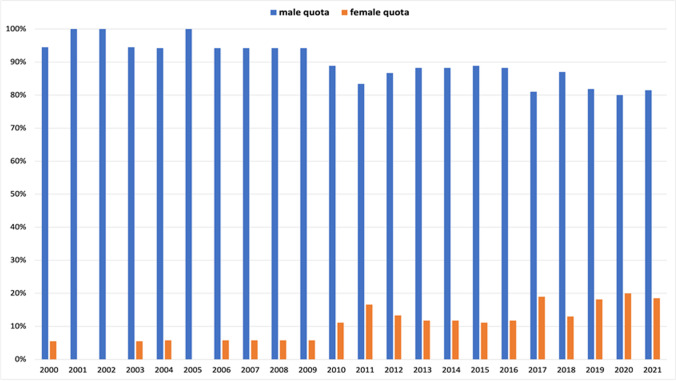
Gender quota of the editorial board of each year. Data are presented in a bar chart, with the percentage of women in orange and the percentage of men in blue

It is noticeable that in 2001, 2002, and 2005, not a single woman was represented on the editorial board (Fig. 11), although women were both first authors and senior authors at this time (see Figs. 3, 4, 5, 6, and 7). In the other years until to 2009, the proportion of women stagnated at around 6% (1 out of 17), markedly contrasting to the increase in female authorship (see Figs. 3, 4, 5, 6, and 7). From 2010 onwards, the proportion of women increased and remained at a level of around 12% (2 out of 17) until 2016. In 2017, there was an upswing to 19% (4 out of 21), which achieved a maximum of 20% (5 out of 25) women on the editorial board in 2020.

### Advisory editor analysis

**Fig. 12:**
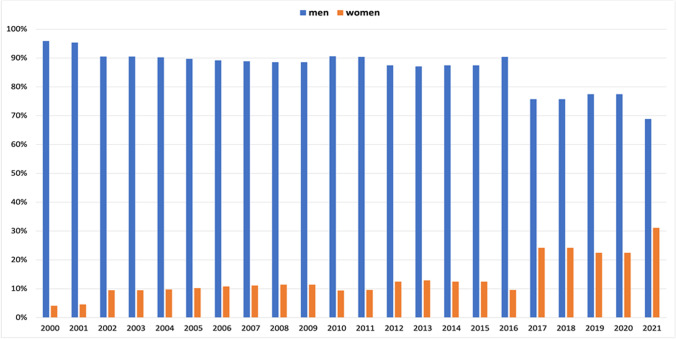
Gender quota of the advisory editors of each year. Data are presented in a bar chart, with the percentage of women in orange and the percentage of men in blue

In 2000, the advisory editors group started with a female representation of just 4% (2 out of 48), but this slowly increased in subsequent years (Fig. 12). The female share hovered around 10% until 2016. In the following 2 years, the percentage of women advisory editors increased to 31% (14 out of 45) in 2021.

## Discussion

### No increase in female author participation in Naunyn–Schmiedeberg’s Archives of Pharmacology over the last decade

The main finding of this analysis is that the proportion of women as authors in *Naunyn–Schmiedeberg’s Archives of Pharmacology* increased moderately from 2000 to 2010 but then stagnated, regardless of whether 2-year- or 5-year intervals, first or senior authorships, or various cities in different geographic locations of Germany with different microcultures are considered (Figs. 3, 4, 5, 6, 7, 8, 9, and 10). In fact, in one city (Hannover), the female share substantially decreased over the past 15 years (Fig. 10), being at the same level as in the year 2000. Furthermore, the share of women in first authorships is more than twice as high as in senior authorships across the entire time analyzed (Figs. 5, 6, 7, and 8). This result may indicate that the percentage of women in leading positions in pharmacological institutes in Germany has not changed much from 2000 to 2020, but there is no official head count.

The proportion of women in the position of first author is substantially higher than the general proportion of women in publications (Figs. 3 and 5). In fact, in one time period dating back already more than 10 years (2009–2010), the female share in first authorships surpassed even 40%, but decreased since then. These data suggest that many women continue to leave research after obtaining their doctorate or after a short postdoc and that the conditions for women for a research career in pharmacology in Germany have, in fact, become less attractive, despite various political actions at multiple levels (see references discussed below).

### Comparison of Naunyn–Schmiedeberg’s Archives of Pharmacology with the global research situation

A recent analysis by BMJ Global Health in May 2020 examined all publications to date on COVID-19 and reported that just one-third of the authors were women. Far fewer women, however, occupied the positions of first and senior author. Since papers on COVID-19 had a high impact on research and society, the projects were commissioned and overseen by researchers in senior positions, which are still largely occupied by men (Pinho-Gomes, A et al. (2020), “Where are the women?”, BMJ Global Health, http://dx.doi.org/10.1136/bmjgh-2020-002922). Another reason may be that women basically had less time than men during the pandemic, since they did most of the domestic work and childcare (World Economic Forum (2021), *Global Gender Gap Report 2021*, http://www3.weforum.org/docs/WEF_GGGR_2021.pdf.).

In terms of the proportion of first authorship and senior authorship, our data fit very well with a recent but much less comprehensive and less systematic analysis in *Nature* (Nature. 2018 Jun;558(7710):344. https://doi.org/10.1038/d41586-018-05465-7. PMID: 29,925,975). In both studies, female authorship in general is about 30%, and senior authorship is about 15%. Thus, in comparison with other analyses, the gender ratio in *Naunyn–Schmiedeberg’s Archives of Pharmacology* maps the general gender structure in research quite well. This suggests that our study is representative for the global situation in biomedical research.

In 2018, the European Union (EU) published that the percentage of women in the medical sciences in the EU exceeds 40%. In Germany, in 2015, the quota of women in the field of natural sciences was 32%, and in medical sciences 50% (https://op.europa.eu/de/publication-detail/-/publication/9540ffa1-4478-11e9-a8ed-01aa75ed71a1, last accessed on September 15, 2021).

In order to better compare the results of our present study with in the general German research situation, we also analyzed the current membership list of the DGPT (German Society for Experimental and Clinical Pharmacology and Toxicology). We were able to assign 2229 of a total of 2254 members (98.9%) exactly to one gender using our algorithm. With 894 women in the DGPT, this results in a female quota of 40%, corresponding very well to the above cited numbers. This analysis also clearly shows that the women’s quota of *Naunyn–Schmiedeberg's Archives of Pharmacology* is significantly below the quota of the DGPT. It is unclear whether this is a journal-specific or a subject-specific issue. This journal focuses more on experimental pharmacology than toxicology or clinical pharmacology. Thus, it will be necessary to study other journals with different foci within the broad field of experimental and clinical pharmacology and toxicology for a proper interpretation of our data. It is also possible that female members of the DGPT work in professional positions where they do not publish scientific papers or are professionally passive. Unfortunately, we have no means of gathering this information.

### Why has the proportion of female authors in Naunyn–Schmiedeberg's Archives of Pharmacology stagnated for a decade?

The fact that the proportion of female authors has stagnated (or even decreased in some dimensions) for more than a decade in all dimensions studied (Figs. 3, 4, 5, 6, 7, 8, 9, and 10) came as a real surprise to us. Since many political actions have been taken at various levels during the past 10 years including European Union legislation (https://ec.europa.eu/info/policies/justice-and-fundamental-rights/gender-equality/equal-economic-independence_en, last accessed on August 3, 2021), federal German law (https://www.bundesregierung.de/breg-de/aktuelles/gleiche-chancen-fuer-frauen-und-maenner-423940, last accessed on August 2, 2021), research support systems (https://www.dfg.de/foerderung/grundlagen_rahmenbedingungen/chancengleichheit/massnahmen/index.html, last accessed on August 2, 2021), and local university regulations (https://www.mhh.de/gleichstellung/programme-und-projekte/professorinnen-programm, last accessed on August 2, 2021) to achieve gender equality, we would have expected that the share of female authors in *Naunyn–Schmiedeberg’s Archives of Pharmacology* had increased over the past 10 years. But this was not the case. Thus, multipronged and joint political actions with substantial financial resources at all levels have not translated into an increase in scientific publications, at least in *Naunyn–Schmiedeberg’s Archives of Pharmacology*, by women during the past decade.

Causal relationships cannot be unambiguously assessed here, since authorship of scientific papers (Figs. 3, 4, 5, 6, 7, 8, 9, and 10), in contrast to the appointment of a person to an editorial board (Figs. 11 and 12; see “Discussion” below), does not proceed in a straight line, but is the result of a multilayered interplay of various parameters.

A factor contributing to our results is certainly the inherent high pressure of the current academic system in Germany to “publish or perish” and to obtain as much grant support as possible to demonstrate “excellence” (https://www.swr.de/swr2/wissen/publish-or-perish-publizieren-in-der-wissenschaft-swr2-wissen-2021-02-06-100.html, last accessed on August 2, 2021). In fact, women tend to avoid competition (Niederle, Vesterlund, M. N. L. V. (2007). *Do women shy away from competition? Do men compete too much?* https://web.standford.edu. https://web.stanford.edu/~niederle/Niederle.Vesterlund.QJE.2007.pdf). In addition to publication pressure in the institutes and the working conditions, university policies, career opportunities, salary expectations, endogenous personal preferences, and gender stereotypes may also play a role. Moreover, family planning occurs exactly at the time when young researchers are making a name for themselves in research and need to push and shape their careers (https://www.sueddeutsche.de/karriere/gender-pay-gap-der-zeitpunkt-ist-unguenstig-fuer-ein-kind-1.3694382, last accessed on August 2, 2021). Women who decide to have children are therefore overtaken by men at this time.

### Positive development of the editorial board: a simple explanation

For editorial board members and advisory editors, there is a positive trend in female participation since 2016 (Figs. 11 and 12). The editorial board and advisory editors are selected at the discretion of the editor-in-chief. Thus, changes in these domains are directly linked to a single-person decision. As a result, changes in female participation become rapidly visible. However, there are limitations for the editor-in-chief in terms of achieving more gender equity because not so rarely, women decline to accept offers or step down from positions.

### Why submissions from Germany to Naunyn–Schmiedeberg’s Archives of Pharmacology decline

The reason for the strong decrease of published papers from German institutes (Fig. 10) is most likely due to the increasing pressure to publish in so-called “high-impact factor journals” such as *Nature*, *Science* and *Cell* for career promotion and obtaining research funding (https://www.swr.de/swr2/wissen/publish-or-perish-publizieren-in-der-wissenschaft-swr2-wissen-2021-02-06-100.html, last accessed on August 2, 2021). *Naunyn–Schmiedeberg’s Archives of Pharmacology* is a solid scientific journal with an impact factor of 3.000 in 2021, but for a high-profile scientific career in Germany, this impact factor is deemed “too low” by many decision makers in science (https://www.forschung-und-lehre.de/absurde-mess-manie-136/; last accessed August 2, 2021). In addition, *Naunyn–Schmiedeberg’s Archives of Pharmacology* is a general pharmacological journal, but there is a clear trend that researchers prefer to publish papers within their specialized research community journals (see, e.g., https://journals.lww.com/behaviouralpharm/pages/default.aspx; https://journals.lww.com/cardiovascularpharm/pages/default.aspx; both last accessed on August 2, 2021).

The editor-in-chief has been actively trying to recruit submissions from both male and female researchers in Germany during the past 5 years by offering rapid, constructive, and fair peer review, with little success so far. The inability of the editor-in-chief to stop the sharp decline in submissions from Germany to *Naunyn–Schmiedeberg’s Archives of Pharmacology*, being the official organ of the Deutsche Gesellschaft für Experimentelle und Klinische Pharmakologie und Toxikologie, probably reflects the strong dominance of the “high-impact factor culture” for a successful career in academic pharmacology in Germany.

### Limitations of our study

We are fully aware of the limitations of our study. We studied just one journal within one given scientific field and a limited time. In addition, we studied only one country (one culture). Future studies should aim to analyze other pharmacological journals, other countries, and longer time periods. In a broader perspective, other biomedical research areas and clinical specialties should of course also be analyzed.

## Five important points to be considered for future studies on this topic

We started this study quite naïvely, expecting it to be simple and straightforward. But this was not the case at all (see “Materials and methods”), and we encountered several unexpected difficulties in different dimensions. Thus, future studies on this topic should be aware of the following five important points:

1. First names in several cultures are unisex. Accordingly, in countries with a traditionally large proportion of international researchers such as the USA (https://www.forschung-und-lehre.de/politik/die-usa-sind-weltweit-das-wichtigste-ziel-fuer-wissenschaftler-829/, last accessed on August 2, 2021), an analysis of the type performed in this study is much more difficult to realize than for Germany.
2. It will also become more difficult to analyze periods prior to the year 2000 because Internet searches become less complete and only favor the identification of established researchers (probably with a bias towards male researchers). Even a comprehensive recourse to the year 2000 was a major challenge.
3. The lack of full disclosure of first names in many journals constitutes a major drawback for such studies. Accordingly, journals are strongly encouraged to publish the full names of authors.
4. Sophisticated detective skills are required here. In our case, we were only able to determine the full first names of many scientists with abbreviated first names through a very time-consuming search. This detective work was only successful because we received support from several quarters, e.g., learned societies, secretaries of research institutes, individual researchers, and historians. A close-meshed network of personal and social relationships is therefore very important for such studies. This makes studies at the regional or national level more feasible, but studies at the international level more difficult.
5. Data protection laws will make it increasingly difficult to access personal databases. Similarly, full disclosure of source data to the public is impossible. For this reason, we do not disclose any individual names of researchers analyzed in this study.

## The more data, the better

Despite the inherent limitations and problems of this work, we hope that this study provides a suitable template for objective analysis of gender participation in the biomedical sciences. As always in science, the more data we have, the better conclusions we can draw and act accordingly.

## Supplementary Information

Below is the link to the electronic supplementary material.

Supplementary file1 (DOCX 48 KB)

## Data Availability

Due to federal data protection laws, we cannot publish the list of full names of authors and editorial board members and advisory editors analyzed in this paper. Neither can we publish the database of *Springer Nature*, as this also contains additional confidential information. Moreover, we are not allowed to provide the list of names of previous and current members of the *Deutsche Gesellschaft für Experimentelle und Klinische Pharmakologie*. However, the numbers underlying the data shown in Figs. 1, 2, 3, 4, 5, 6, 7, 8, 9, 10, 11, and 12 are provided as supplemental material.

