# Supplementary material for: Gender-specific analysis of the authors and the editorial board of Naunyn–Schmiedeberg’s Archives of Pharmacology from 2000 to 2020
Source: Naunyn Schmiedebergs Arch Pharmacol. 2021 Oct 8;395(1):39–50. doi: 10.1007/s00210-021-02166-3 (PMC8497184; doi:10.1007/s00210-021-02166-3)
Supplement: Supplementary file 1 — Supplementary file1 (DOCX 48 KB) [file 210_2021_2166_MOESM1_ESM.docx]

**Supplementary Source Data**

**Gender-specific analysis of the authors and the editorial board of *Naunyn-Schmiedeberg's Archives of Pharmacology* from 2000-2020**

**Rebecca Zehetbauer, Florentin von Haugwitz, Roland Seifert**

**Source Data to Figure 1 and Figure 2**

| **Gender** | **Number** | **Percentage of determinables** | **Percentage** |
| --- | --- | --- | --- |
| Men | 2,071 | 71.76 % |  |
| Women | 815 | 28.24 % |  |
| Determinable | 2,886 |  | 98.57 % |
| Undeterminable | 42 |  | 1.43 % |
| In total | 2,928 |  |  |

**Source Data to Figure 3, Figure 4, Figure 5, Figure 6, Figure 7, Figure 8 and Figure 10**

| **Year** | **Number** | **Gender** | **Total** | **Percentage** | **Number of papers** | **Female first authors** | **Female senior authors** |
| --- | --- | --- | --- | --- | --- | --- | --- |
| 2020 | 64 | M | 87 | 73.56 % | 24 | 2 | 5 |
|  | 23 | F |  | 26.44 % |  |  |  |
| 2019 | 44 | M | 70 | 62.86 % | 17 | 14 | 1 |
|  | 26 | F |  | 37.14 % |  |  |  |
| 2018 | 58 | M | 84 | 69.05 % | 21 | 7 | 3 |
|  | 26 | F |  | 30.95 % |  |  |  |
| 2017 | 82 | M | 116 | 70.69 % | 24 | 6 | 4 |
|  | 34 | F |  | 29.31 % |  |  |  |
| 2016 | 60 | M | 88 | 68.18 % | 19 | 8 | 4 |
|  | 28 | F |  | 31.82 % |  |  |  |
| 2015 | 80 | M | 116 | 68.97 % | 28 | 11 | 3 |
|  | 36 | F |  | 31.03 % |  |  |  |
| 2014 | 96 | M | 139 | 69.06 % | 36 | 13 | 4 |
|  | 43 | F |  | 30.94 % |  |  |  |
| 2013 | 92 | M | 132 | 69.70 % | 29 | 8 | 1 |
|  | 40 | F |  | 30.30 % |  |  |  |
| 2012 | 85 | M | 142 | 59.86 % | 30 | 15 | 7 |
|  | 57 | F |  | 40.14 % |  |  |  |
| 2011 | 107 | M | 155 | 69.03 % | 38 | 11 | 5 |
|  | 48 | F |  | 30.97 % |  |  |  |
| 2010 | 111 | M | 167 | 66.47 % | 28 | 14 | 4 |
|  | 56 | F |  | 33.53 % |  |  |  |
| 2009 | 89 | M | 125 | 71.20 % | 27 | 9 | 3 |
|  | 36 | F |  | 28.80 % |  |  |  |
| 2008 | 77 | M | 127 | 60.63 % | 26 | 9 | 6 |
|  | 50 | F |  | 39.37 % |  |  |  |
| 2007 | 98 | M | 132 | 74.24 % | 26 | 9 | 3 |
|  | 34 | F |  | 25.76 % |  |  |  |
| 2006 | 83 | M | 119 | 69.75 % | 29 | 10 | 6 |
|  | 36 | F |  | 30.25 % |  |  |  |
| 2005 | 107 | M | 146 | 73.29 % | 33 | 9 | 5 |
|  | 39 | F |  | 26.71 % |  |  |  |
| 2004 | 150 | M | 204 | 73.53 % | 40 | 18 | 2 |
|  | 54 | F |  | 26.47 % |  |  |  |
| 2003 | 129 | M | 171 | 75.44 % | 36 | 9 | 4 |
|  | 42 | F |  | 24.56 % |  |  |  |
| 2002 | 113 | M | 150 | 75.33 % | 36 | 9 | 3 |
|  | 37 | F |  | 24.67 % |  |  |  |
| 2001 | 180 | M | 224 | 80.36 % | 52 | 9 | 2 |
|  | 44 | F |  | 19.64 % |  |  |  |
| 2000 | 166 | M | 192 | 86.46 % | 52 | 8 | 2 |
|  | 26 | F |  | 13.54 % |  |  |  |

**More specific source data to Figure 3**

| Time  period | Male  quota | Female quota |  | Time  period | In total | Men | Women |
| --- | --- | --- | --- | --- | --- | --- | --- |
| 2000 | 86.55 % | 13.45 % |  | 2000 | 192 | 166 | 26 |
| 2001-2002 | 77.85 % | 22.15 % |  | 2001-2002 | 374 | 293 | 81 |
| 2003-2004 | 74.49 % | 25.51 % |  | 2003-2004 | 375 | 279 | 96 |
| 2005-2006 | 71.52 % | 28.48 % |  | 2005-2006 | 265 | 190 | 75 |
| 2007-2008 | 67.43 % | 32.57 % |  | 2007-2008 | 259 | 175 | 84 |
| 2009-2010 | 68.84 % | 31.16 % |  | 2009-2010 | 292 | 200 | 92 |
| 2011-2012 | 64.45 % | 35.55 % |  | 2011-2012 | 297 | 192 | 105 |
| 2013-2014 | 69.38 % | 30.62 % |  | 2013-2014 | 271 | 188 | 83 |
| 2015-2016 | 68.57 % | 31.43 % |  | 2015-2016 | 204 | 140 | 64 |
| 2017-2018 | 69.87 % | 30.13 % |  | 2017-2018 | 200 | 140 | 60 |
| 2019-2020 | 68.21 % | 31.79 % |  | 2019-2020 | 157 | 108 | 49 |

**More specific source data for Figure 4**

| Time period | Percentage of Men | Percentage of Women |
| --- | --- | --- |
| 2000-2005 | 77.40 % | 22.60 % |
| 2006-2010 | 68.45 % | 31.55 % |
| 2011-2015 | 67.32 % | 32.68 % |
| 2016-2020 | 68.86 % | 31.14 % |

**Absolute numbers for Figure 4**

| Time period | In total | Men | Women |
| --- | --- | --- | --- |
| 2000-2005 | 1087 | 845 | 242 |
| 2006-2010 | 670 | 458 | 212 |
| 2011-2015 | 684 | 460 | 224 |
| 2016-2020 | 445 | 308 | 137 |

**More specific source data for Figure 5**

| First Authorship | | | | | | | |
| --- | --- | --- | --- | --- | --- | --- | --- |
| Time  period | Men | Women |  | Time  period | In total | Men | Women |
| 2000 | 84.62 % | 15.38 % |  | 2000 | 52 | 44 | 8 |
| 2001-2002 | 79.55 % | 20.45 % |  | 2001-2002 | 88 | 70 | 18 |
| 2003-2004 | 64.47 % | 35.53 % |  | 2003-2004 | 76 | 49 | 27 |
| 2005-2006 | 69.35 % | 30.65 % |  | 2005-2006 | 62 | 43 | 19 |
| 2007-2008 | 65.38 % | 34.62 % |  | 2007-2008 | 52 | 34 | 18 |
| 2009-2010 | 58.18 % | 41.82 % |  | 2009-2010 | 55 | 32 | 23 |
| 2011-2012 | 61.76 % | 38.24 % |  | 2011-2012 | 68 | 42 | 26 |
| 2013-2014 | 67.69 % | 32.31 % |  | 2013-2014 | 65 | 44 | 21 |
| 2015-2016 | 59.57 % | 40.43 % |  | 2015-2016 | 47 | 28 | 19 |
| 2017-2018 | 71.11 % | 28.89 % |  | 2017-2018 | 45 | 32 | 13 |
| 2019-2020 | 60.98 % | 39.02 % |  | 2019-2020 | 41 | 25 | 16 |

**More specific source data for Figure 6**

| Time period | Percentage of Men | Percentage of Women |
| --- | --- | --- |
| 2000-2005 | 75.10 % | 24.90 % |
| 2006-2010 | 62.50 % | 37.50 % |
| 2011-2015 | 63.98 % | 36.02 % |
| 2016-2020 | 64.76 % | 35.24 % |

**Absolute numbers for Figure 6**

| Time period | In total | Men | Women |
| --- | --- | --- | --- |
| 2000-2005 | 249 | 187 | 62 |
| 2006-2010 | 136 | 85 | 51 |
| 2011-2015 | 161 | 103 | 58 |
| 2016-2020 | 105 | 68 | 37 |

**More specific source data for Figure 7**

| Senior Authorship | | | | | | | |
| --- | --- | --- | --- | --- | --- | --- | --- |
| Time period | Men | Women |  | Time  period | In total | Men | Women |
| 2000 | 96.15 % | 3.85 % |  | 2000 | 52 | 50 | 2 |
| 2001-2002 | 94.32 % | 5.68 % |  | 2001-2002 | 88 | 83 | 5 |
| 2003-2004 | 92.11 % | 7.89 % |  | 2003-2004 | 76 | 70 | 6 |
| 2005-2006 | 82.26 % | 17.74 % |  | 2005-2006 | 62 | 51 | 11 |
| 2007-2008 | 82.70 % | 17.30 % |  | 2007-2008 | 52 | 43 | 9 |
| 2009-2010 | 87.28 % | 12.72 % |  | 2009-2010 | 55 | 48 | 7 |
| 2011-2012 | 82.36 % | 17.64 % |  | 2011-2012 | 68 | 56 | 12 |
| 2013-2014 | 92.31 % | 7.69 % |  | 2013-2014 | 65 | 60 | 5 |
| 2015-2016 | 85.11 % | 14.89 % |  | 2015-2016 | 47 | 40 | 7 |
| 2017-2018 | 84.45 % | 15.55 % |  | 2017-2018 | 45 | 38 | 7 |
| 2019-2020 | 85.37 % | 14.63 % |  | 2019-2020 | 41 | 35 | 6 |

**More specific source data for Figure 8**

| Time period | Percentage of men | Percentage of women |
| --- | --- | --- |
| 2000-2005 | 92.77 % | 7.23 % |
| 2006-2010 | 83.82 % | 16.18 % |
| 2011-2015 | 87.58 % | 12.42 % |
| 2016-2020 | 83.81 % | 16.19 % |

**Absolute numbers for Figure 8**

| Time period | In total | Men | Women |
| --- | --- | --- | --- |
| 2000-2005 | 249 | 231 | 18 |
| 2006-2010 | 136 | 114 | 22 |
| 2011-2015 | 161 | 141 | 20 |
| 2016-2020 | 105 | 88 | 17 |

**Source data for Figure 9**

| Cities | | | | |
| --- | --- | --- | --- | --- |
| Time period | Institutes | Female Quota | In total | Women |
| 2000-2005 | Hannover | 33.33 % | 3 | 1 |
|  | Heidelberg | 20.20 % | 99 | 20 |
|  | Bonn | 20.00 % | 55 | 11 |
| 2006-2010 | Hannover | 38.46 % | 39 | 15 |
|  | Heidelberg | 27.27 % | 99 | 27 |
|  | Bonn | 32.72 % | 55 | 18 |
| 2011-2015 | Hannover | 36.93 % | 111 | 41 |
|  | Heidelberg | 36.95 % | 92 | 34 |
|  | Bonn | 42.30 % | 52 | 22 |
| 2016-2020 | Hannover | 30.50 % | 59 | 18 |
|  | Heidelberg | 35.29 % | 51 | 18 |
|  | Bonn | 29.03 % | 31 | 9 |

**Source Data for Figure 11**

| Editorial Board | | | | |
| --- | --- | --- | --- | --- |
| Year | In total | Men | Women | Female Quota |
| 2000 | 18 | 17 | 1 | 5.50 % |
| 2001 | 18 | 18 | 0 | 0.00 % |
| 2002 | 17 | 17 | 0 | 0.00 % |
| 2003 | 18 | 17 | 1 | 5.50 % |
| 2004 | 17 | 16 | 1 | 5.80 % |
| 2005 | 17 | 17 | 0 | 0 % |
| 2006 | 17 | 16 | 1 | 5.80 % |
| 2007 | 17 | 16 | 1 | 5.80 % |
| 2008 | 17 | 16 | 1 | 5.80 % |
| 2009 | 17 | 16 | 1 | 5.80 % |
| 2010 | 18 | 16 | 2 | 11.11 % |
| 2011 | 18 | 15 | 3 | 16.60 % |
| 2012 | 15 | 13 | 2 | 13.30 % |
| 2013 | 17 | 15 | 2 | 11.76 % |
| 2014 | 17 | 15 | 2 | 11.76 % |
| 2015 | 18 | 16 | 2 | 11.11 % |
| 2016 | 17 | 15 | 2 | 11.76 % |
| 2017 | 21 | 17 | 4 | 19.00 % |
| 2018 | 23 | 20 | 3 | 13.00 % |
| 2019 | 22 | 18 | 4 | 18.18 % |
| 2020 | 25 | 20 | 5 | 20 % |
| 2021 | 27 | 22 | 5 | 18.51 % |

**Source Data for Figure 12**

| Advisory Editors | | | | |
| --- | --- | --- | --- | --- |
| Year | In total | Men | Women | Female Quota |
| 2000 | 48 | 46 | 2 | 4.10 % |
| 2001 | 43 | 39 | 2 | 4.60 % |
| 2002 | 42 | 38 | 4 | 9.50 % |
| 2003 | 42 | 38 | 4 | 9.50 % |
| 2004 | 41 | 37 | 4 | 9.75 % |
| 2005 | 39 | 35 | 4 | 10.25 % |
| 2006 | 37 | 33 | 4 | 10.81 % |
| 2007 | 36 | 32 | 4 | 11.11 % |
| 2008 | 35 | 31 | 4 | 11.42 % |
| 2009 | 35 | 31 | 4 | 11.42 % |
| 2010 | 32 | 29 | 3 | 9.37 % |
| 2011 | 31 | 28 | 3 | 9.60 % |
| 2012 | 32 | 28 | 4 | 12.50 % |
| 2013 | 31 | 27 | 4 | 12.90 % |
| 2014 | 32 | 28 | 4 | 12.50 % |
| 2015 | 32 | 28 | 4 | 12.50 % |
| 2016 | 31 | 28 | 3 | 9.60 % |
| 2017 | 33 | 25 | 8 | 24.24 % |
| 2018 | 33 | 25 | 8 | 24.24 % |
| 2019 | 40 | 31 | 9 | 22.50 % |
| 2020 | 40 | 31 | 9 | 22.50 % |
| 2021 | 45 | 31 | 14 | 31.11 % |
